# Prevalence of myopia among secondary school students in Welkite town: South-Western Ethiopia

**DOI:** 10.1186/s12886-020-01457-2

**Published:** 2020-05-04

**Authors:** Sara Abebaw Gessesse, Addisu Worku Teshome

**Affiliations:** Department of Ophthalmology, College of Health Science, School of Medicine, Addis Ababa University, Post Box: 31531, Addis Ababa, Ethiopia

**Keywords:** Myopia, Visual acuity, Non-cycloplegic refraction, Secondary school student

## Abstract

**Background:**

Refractive error, especially myopia, is the most common eye disorder in the world and a significant cause of correctable visual impairment. The aim of this study was to assess the prevalence of myopia among secondary school students in Welkite town, South-Western Ethiopia.

**Methods:**

A school based cross sectional study was carried out among secondary school students of 13 to 26 years of age. The students were selected using a multi stage-stratified cluster sampling technique from four secondary schools. The students’ socioeconomic background, usage of eyeglasses and parental myopia was assessed by a questionnaire before visual acuity assessment. Students with visual acuity of less than or equal to 6/12 in the worse eye, who showed vision improvement with pinhole, underwent non-cycloplegic retinoscopy and subjective refraction. Myopia was defined as a spherical equivalent of less than or equal to − 0.50 diopters. Logistic regression was used to see the association of myopia with age, sex, grade level, ethnicity, parental history of myopia and severity.

**Results:**

A total of 1271 students with a response rate of 89.4% were evaluated. The mean age was 16.56+ 1.51 years. Eighty three students were identified to have myopic refractive error making the prevalence of 6.5% (95% CI: 5.30, 8.02). Of 648 females, 50 (7.7%) had myopia while 33 (5.3%) of 623 males had myopia making females relative risk to be 1.5 times that of males. From the total students diagnosed to have refractive error (n = 92), myopia constituted 83/92 (90.2%) of the students indicating that it is the commonest type of refractive error found amongst secondary school students. Only 36.1% of students with myopia wore eyeglasses when they attended the survey. Myopia was more common among older age group 17–21 years (OR: 1.54 95% CI 0.986–2.415) and higher grade level 11–12 (OR: 1.14 95% CI 0.706–1.847).

**Conclusions:**

The prevalence of myopia is high in our study. Attention to the correction of myopia in secondary schools students of Welkite town using eyeglasses can prevent a major proportion of visual impairment.

## Background

Myopia is the optical condition of the nonaccommodating eye in which parallel rays of light entering the eye are brought to a focus anterior to the retina. In the average ophthalmic practice, myopes represent an even larger proportion of patients because of their lifelong dependence on visual aids [1].

Genetic and environmental factors are well known components that contribute to the development of myopia [2]. During the teenage years, when the body grows rapidly, myopia may become worse [3]. Myopia has been associated with parental myopia, socio-economic status, intelligence and educational level, and the exposure to near work [4–9]. School children are considered a high risk group because uncorrected refractive error can seriously affect their learning abilities, physical and mental development [10].

The prevalence of myopia varies with age, race and gender, ranging from 0.79% in rural Nepal from Refractive Error Study in Children [10] to 79.9% in Guangzhou, China among subjects aged 15 years [11]. Jordan reported a prevalence of 17.6%, among secondary school students aged 12 to 17 years [12]. A prevalence of 5.8% was reported from rural Mongolia [13]. In Africa, a prevalence of 5.6% was found in Tanzania [14] and 7.5% from Debark and Kola Diba in Ethiopia that included 1134 pre-school and school children aged 5 to 15 years [15].

Importantly, uncorrected myopia is a significant cause of visual disability in children and adults. Many students with poor vision due to myopia remain undiagnosed and perform poorly in school. This study was conducted to assess the prevalence of myopia in secondary school students (grade 9–12) in Welkite town of South West Ethiopia.

## Methods

The education system in Ethiopia is divided into three divisions consisting of primary school, lower secondary school, and higher secondary school. The primary schooling lasts for 8 years, the lower secondary 2 years, and the higher secondary 2 years. Primary schooling is compulsory for children between the ages of 6 and 14—grades one through eight.

This school based cross-sectional study was conducted in Welkite town from April 1 to 30, 2016. Welkite is a zonal town in South-Western Ethiopia [16]. The town has four secondary schools. Two schools have grade 9–12, one with grade 9 and 10, and one school has only grade 9. There were a total of 6182 secondary school students of which 3308 were males and 2824 were females. The population consisted of all secondary schools in the town, number of students in grade 9 were 2084; in grade 10, 2122; in grade 11, 921; and in grade 12, 885.

We sampled Grades 9 through 12 students of the four secondary schools using a multi staged-stratified cluster sampling technique based on proportional sample allocation in respect to their population size. The sample size was calculated with a prevalence rate of 7.5% for myopia which was taken from a study done in Debark and Kola Diba towns [15], North West Ethiopia with a 2% error rate and a 95% confidence interval. The cluster design effect and non-response rate were assumed to be 2 and 5%, respectively. The calculated sample size was 1422. All the students attending these four secondary schools were eligible to participate in the study.

The study adhered to the tenets of the Declaration of Helsinki and was approved by Research and Publication Committee of the Department of Ophthalmology, Collage of Health Sciences, Addis Ababa University. Permission was requested by formal letter written from the department of ophthalmology detailing the study objectives and procedures, and consent was obtained from the local Education Service Authority and Education Bureau for conducting the study in the secondary schools.

Authorities of secondary schools, heads and students of selected classes were briefed on the study purpose and procedures before beginning the study. Parents or guardians of students were informed about the aim and study procedures through their children and an information sheet along with an informed consent form. They were asked to send the written informed consent for allowing their children to participate in the study. If the parents or guardians of the student did not return the signed informed consent form on the examination day, the student was not enrolled in the study. Interview and eye examinations were carried out in the presence of an assigned data collection facilitator from each school. We ensured that student’s written informed consent was obtained from those students 18 years and older willing to participate in the study for evaluation, in order to analyze, summarize and publish those data.

To assure data quality, the work was carried out by a team that comprised an ophthalmologist, ophthalmic nurse, optometrist and one person from each school who facilitated data collection. The whole study was conducted in school premises during the school days and school hours. Pre-testing was conducted on 5% of students of other sections which were not included in actual study. A questionnaire, participant information sheet and written informed consent were administered in the student’s preferred language (Guragigna, Amharic, Oromifaa or English). A standardized questionnaire, which was modified from the Sydney Myopia Study group [17], was adapted and used in this study. For each eligible student, the questionnaire included items to obtain data on the socio-demographic background such as age, sex, ethnicity, usage of eye glasses and information on parental myopia. The ophthalmic examinations included distance visual acuity measurement with and without pinhole, non- cycloplegic retinoscopic refraction, subjective refraction, ocular alignment and motility evaluation, and, anterior segment and fundus examination.

An ophthalmic nurse measured visual acuity for each eye using the Snellen‘s E -chart hanging on the wall at a distance of 6 m in a well-lighted classroom. A line of optotypes is generally considered to have been read correctly when more than half of the optotypes presented have been read correctly on the chart. Visual acuity was repeated with pinhole. Students with uncorrected and presenting visual acuity of less than or equal to 6/12, whose vision improvement showed with pinhole were refracted by an optometrist using streak retinoscope and trial lens. Non- cycloplegic retinoscopic refraction was performed in darkened room by maintaining 2/3 m distance from the examiner. For those with positive retinoscopic refraction, subjective refraction was performed using standard refraction trial set and frame, and eye glasses were prescribed.

All students with visual acuity of less or equal to 6/12 and whose visual acuity did not improve with pinhole were evaluated by the ophthalmologist (SAG) using torch light, portable slit lamp biomicroscopy and post dilation direct ophthalmoscopy by 1% Tropicamide eye drop in order not to miss those students with retinal finding suggestive of high myopia. Those students with refractive error were advised to have regular follow- up at a nearby eye care center. Some students with other eye abnormalities of position and motility, the anterior segment, the optic disc, the macula or combination requiring further management were referred to an appropriate ophthalmic center.

For the purpose of this study, the following operational definitions were used. The spherical equivalent of the refractive error was calculated as the spherical refractive error plus half of the minus cylindrical value obtained. Prescribing eye glasses using subjective refraction was based on accepting lowest minus lens that lead to measurable visual improvement and highest plus lens not lead to measurable visual reduction. The worse eye was defined as the eye with the greater absolute value of the spherical equivalent of the subjective refraction. Myopia was defined as visual acuity of less than or equal to 6/12 in the worse eye that can be corrected by a spherical equivalent of − 0.5 diopter (D) or worse [10, 17]. Mild myopia defined as less than or equal to − 0.50 and greater than − 3.0 D; moderate myopia defined as less than or equal to − 3.0 D and greater than − 6.0 D; and sever myopia defined as less than or equal to − 6.0 D [18].

Data were cleaned, coded and entered to the statistical package for social science (SPSS, version 20, IBM, Chicago) for analysis. Descriptive analysis was applied to describe the variables of the study. The continuous variables were represented as mean + standard deviation and categorical variables were represented as percentage. The subjective refraction results were used for the analyses of visual correction in the worse eye. Univariate analysis and logistic regression were used to see the association of myopia with relevant variables such as age, sex, grade level, ethnicity, parental history, and severity. An odds ratio was computed for each explanatory variable to determine the strength of association with outcome variable and to control the effect of confounding factors. *P* value < 0.05 was considered statistically significant.

## Results

Of the total 1422 secondary school students sampled, 1271 participants aged 13–26 years old, comprising 648 (51.0%) females and 623 (49.0%) males were included in the final analysis of study with a response rate of 89.4%. The mean age for males was 16.7 (± 1.62) and females, 16.4 (± 1.37) years. Most of the study participants 695 (54.7%) were aged 13–16 years old; and 920 (72.4%) belong to Gurage ethnic group. The largest numbers of participants were grade 9 students 469 (36.9%) (Table 1).

**Table 1:** Socio-demographic background of the study participants attending secondary schools in Welkite town, April 2016, (n = 1271)

Variable	Category	Number	%
Age in years	13	6	0.5
	14	51	4.0
	15	275	21.6
	16	363	28.6
	17	227	17.9
	18	238	18.7
	19	67	5.3
	20	33	2.6
	21	5	0.4
	22	3	0.2
	23	2	0.2
	26	1	0.1
Sex	Female	648	51.0
	Male	623	49.0
Ethnicity	Gurage	920	72.4
	Amhara	234	18.4
	Oromo	56	4.4
	Tigrea	14	1.1
	Others	47	3.7
Grade level	9	469	36.9
	10	437	34.4
	11	185	14.6
	12	180	14.2
Parental Myopia	Yes	264	20.77
	No	1007	79.23

Of the 1271 students participated in the study 99 (7.8%) had a visual acuity of 6/12 or worse at least in one eye. The underline causes for visual reduction were refractive error 92 (93%), amblyopia 4 (4%), corneal problem 2 (2%) and lens problem 1 (1%). Among the students with refractive error, myopia constituted 83 (90.2%) of the students. Moderate visual impairment (presenting vision <  6/18–6/60 in the worse eye) was present in 54 (65.1%) students with myopia. In this study 6.5% of secondary school participant students had myopic refractive error (Table 2).

**Table 2:** Distribution of uncorrected visual acuity among secondary school students with myopia, Welkite town (n = 83)

WHO vision category	Uncorrected visual acuity	Right Eye	Left Eye	Both Eyes	Total n (%)
Mild visual impairment	6/12 to 6/18	3	7	13	23 (27.7%)
Moderate visual impairment	< 6/18 to 6/60	11	14	29	54 (65.1%)
Severe visual impairment	< 6/60 to 3/60	–	2	4	6 (7.2%)
Total		14	23	45	83 (100%)

The mean spherical equivalent of myopic refractive error was − 3.6 D in the right eye (SD = 3.4: range − 0.75 D to − 13.00 D) and − 4.1 D in the left eye (SD = 4.1: range − 0.50 D to − 14.0 D). Only 30 (36.1%) of students with myopia wore eyeglasses when they attended the survey.

Table 3 shows the distribution of myopia among ethnic groups, myopia was found in 3 (5.4%) in Oromo, 59 (6.4%) in Gurage, 1 (7.1%) in Tigrea, 17 (7.3%) in Amhara and 3 (6.4%) in others.

**Table 3:** Distribution of myopia among secondary school students ethnic groups, Welkite town

Ethnic Group	Number (%)	Prevalence of Myopia	Relative risk
Gurage	920 (72.4%)	59 (6.4%)	1.2
Amhara	234 (18.4%)	17 (7.3%)	1.4
Oromo	56 (4.4%)	3 (5.4%)	1.0
Tigrea	14 (1.1%)	1 (7.1%)	1.3
Others	47 (3.7%)	3 (6.4%)	1.2
Total	1271	83	

The prevalence of myopia was higher in females than males, in 18 year old students as compared with other age, in grade 10 participants versus other grade, and in mild myopic versus moderate and severe individuals (Table 4).

**Table 4:** The prevalence of myopia by different sex, age, grade level and severity groups among secondary school students in Welkite town (*n* = 83)

Variable	Category	Number of myopia (%)
Sex	Female	50 (60.2)
	Male	33 (39.8)
Age	14	4 (4.8)
	15	17 (20.5)
	16	16 (19.3)
	17	17 (20.5)
	18	21 (25.3)
	19	5 [6]
	20	3 (3.6)
Grade level	9	21 (25.3)
	10	36 (43.4)
	11	17 (20.5)
	12	9 (10.8)
Severity	Mild	53 (63.9)
	Moderate	10 (12.0)
	Severe	20 (24.1)
Parent	Myopic	50 (60.2)
	Non-myopic	33 (39.8)

A univariate analysis was employed to test the association between relevant variables with myopia. The prevalence of myopia was not associated with sex, and ethnicity. There were larger number of myopic females 50 (60.2%) than males 33 (39.8%), but it was not statistically significant (p = 0.083) (Table 5). Of 648 females, 50 (7.7%) had myopia while 33 (5.3%) of 623 males had myopia making the relative risk of myopia among females to be 1.5 times compared to that of males. The prevalence of myopia was 18.94% in students with myopic parents, where as 3.28% for those students without parental myopia with relative risk of 5.77.

**Table 5:** Univariate analysis of associations between the prevalence of Myopia and associated factors in secondary school student, Welkite town (n = 83)

Variable	OR	95%CI	*p*-value
Sex (Female Versus Male)	1.54	0.95–2.35	0.083
Age groups (17–26 versus 13–16 years)	1.54	0.97–2.42	0.068
Grade level (11–12 versus 9–10)	1.19	0.71–1.85	0.587
Ethnicity (Gurage versus Non Gurage)	1.01	0.61–1.66	0.971
Parental myopia (present verses absent)	6.9	4.34–10.97	0.01

In binary logistic regression the prevalence of myopia as the dependent variable and factors, which were associated with myopia prevalence in the univariate analysis, as independent variables, revealed that students in the age category of 17–21 years were 1.54 times likely to encounter myopia when compared with those in the age category 13–16 years. Students who reside in grade 11–12 were more likely to have myopia when compared to those who were grade 9–10. There was strongly significant association between parental history of myopia and severity (p = 0.01). It was shown that 54.7% of mild myopia, 60% of moderate myopia and 75% of severe myopia had also parental history of myopia (Table 6).

**Table 6:** Linear multivariate regression analysis of the associations between the prevalence of Myopia and associated factors in secondary school student, Welkite town (n = 83)

Variable	Reg. Coef. B	Stand. Coeff. Beta	*p*-value	95% CI
Age	−0.006	- 0.024	0.497	−0.022 - 0.011
Grade	0.006	0.040	0.264	- 0.005 - 0.018
Sex	0.337	2.216	0.145	0.890–2.204
Ethnicity	−0.008	0.000	0.990	0,094–10.400
Parental myopia	−0.382	2.008	0.157	0.403–1.158

## Discussion

Although school screenings have been conducted elsewhere, the study on the prevalence of myopia among secondary school students in Welkite town was the first of its kind. In this study, the prevalence of myopia was found to be 6.5% with visual acuity of 6/12 or less in the worst eye and a spherical equivalent power of − 0.50D or below. The result was comparable with figures reported from Tanzania [14], Debark and Kola Diba town, North West Ethiopia [15] and rural Poland [19].

Myopia prevalence in this study population was considerably lower than in a study population from Beijing [20], Shanghai [21] and rural China [22]. But, it was found to be higher than a study from rural Nepal among 15 years old children [10]. This variation could be due to the difference in genetic back ground, differences in lifestyle, sample size or examination technique used.

In this study, higher prevalence of myopia was associated with higher age and higher grade level which was consistent with the result of a study done in Iran high school which reported similar trend [23]. Ethnicity was not found to have an association with the prevalence of myopia in our study which is contradictory with a study done in China (Non-Han ethnicity had a significantly lower prevalence of myopia and high myopia than students with Han ethnicity) [21]. It has remained unclear whether these ethnic differences in the prevalence of myopia were due to genetic differences or due to differences in lifestyle or differences in the time of study.

In this study, sex had no association with the prevalence of myopia and this was consistent with the study from Shanghai [21] that reported similar result. However, it differs from the study from Debark and Kola Diba town which revealed refractive error to be significantly associated with female sex [15]. The severity assessment has indicated that 63.9% of myopia was mild, 12% moderate and 24.1% severe. The severe form was higher when compared to the Chinese study, 10%, [21], the reason may be that the cause could be more of hereditary in our set up.

Although there are several important findings in this study, our study does have some potential limitations. First, the parental history of myopia was obtained using reported questionnaires. This method was used in previously reported studies, but it could be subject to information bias. Second, the non cycloplegic refraction data collection method was selected by considering the parental or guardian worry about the possible and the potential side effects of cyclopentolate, we decreased the parental or guardian refusal drop out and we also worried about the mydriasis effect of cyclopentolate will affect the students’ learning. Therefore, non cycloplegic refraction might have increased the rate of pseudomyopia. Lastly, we selected one town secondary school students, the sample might have been subjected to selection bias. Future studies are needed on large population of secondary school students to determine the risk factors.

## Conclusions

As a school based survey, this study revealed the prevalence of myopia to be 6.5% among secondary school students aged 13–26 years. Higher prevalence of myopia was significantly associated with higher age and higher grade level. Prevalence of high myopia was higher in students with family history of myopia. Only 36.1% of students with myopia wore eyeglasses when they attended the survey. This study also indicates the need for a regular visual screening program for school children. School screening program can help in early detection and management of myopia and its associated complications. It is recommended those stakeholders or policy makers’ plan or design ways for direct school screening program. Future studies are needed on large population of students to determine the risk factors.

## Data Availability

The datasets used and/or analyzed during in the current study are available from the corresponding author on reasonable request.

## Abbreviations

CI: Confidence Interval
D: Diopters
n: Number
OR: Odds Ratio
Reg. Coeff Beta: Regression Coefficient Beta
Stand. Coeff. Beta: Standard Coefficient Beta
WHO: World Health Organization
